# Are Huddles the Missing PEACE of the Puzzle in Implementing Clinical Innovation for the Eating Disorder and Autism Comorbidity?

**DOI:** 10.3389/fpsyt.2020.593720

**Published:** 2020-11-05

**Authors:** Katherine Amanda Smith, Kate Tchanturia

**Affiliations:** ^1^Department of Psychological Medicine, King's College London, Institute of Psychiatry, Psychology and Neuroscience, London, United Kingdom; ^2^South London and Maudsley National Health Service (NHS) Foundation Trust, National Eating Disorder Service, London, United Kingdom; ^3^Department of Psychology, Illia State University, Tbilisi, Georgia

**Keywords:** huddle, team, multidisciplinary, communication, implementation, autism, eating disorders, innovation

## Abstract

Huddles are brief, time-limited, focused meetings to help organize and support clinical teams. Huddles have demonstrated their value and transferable benefits across a range of settings. Based on their transferable nature, their potential could be unacknowledged as a clinical implementation technique, particularly in specific subgroups of patients with anorexia who need a higher level of care. An innovative clinical pathway aimed at supporting autistic patients with eating disorders (PEACE Pathway) evaluated the use of weekly PEACE huddles for the multidisciplinary team as part of the implementation process across a 12-months period. A total of 283 responses evaluated the huddle as useful on average 84/100. Using content analysis, several perceived benefits were found of the huddles which were in line with the underpinnings of traditional huddles, suggesting that huddles are transferable as implementation techniques, as evidence by a team providing higher-level care for eating disorders.

## Introduction

Huddles can be defined as brief, regular meetings aimed at keeping team members informed, actively evaluating and maintaining procedures, goal setting, and thinking about future directions (1). Huddles are different to other types of team meetings, such as rounds which take place with the patient (2), briefings and debriefings which take place before and after specific events (2). Huddles have demonstrated successes by increasing effective and efficient work, particularly regarding safety, across various professions from healthcare to the military (3, 4). Although most commonly utilized daily or prior to a procedure (5), the use of weekly huddles has been successful, especially with increasing clinician attendance (6, 7). Furthermore, huddles have been found to be the most beneficial when adapted to the demands of the environment they are supporting (8).

Huddles have been used successfully in mental health-related concerns in dementia care with improvements in collaboration, teamwork, support and discussing specific behaviors (9). With such translatable benefits, huddles are potentially a very important implementation strategy when rolling out clinical innovation in mental health services. Identifying and evaluating strategies for implementation increases the chances of successfully implementing clinical innovation. Implementation science is developing theory-based knowledge about implementation techniques and approaches that help roll out an innovation, sustain it, and facilitate scaling up (10). There is an existing body of evidence on integrated care in mental health settings (11) but little evidence on huddles as a clinical implementation tool and why it might be important.

Theoretical underpinnings of huddles suggest that they promote benefits to attendees which include teamwork, communication, education and training, and shared professional identity (12). Teamwork can be defined as understanding competencies and principles that people use to accomplish interdependent work (13). Healthcare settings have acknowledged the role of teamwork in delivering high-quality patient care (14). Care is often collaborative, with different disciplines working together to ensure patients are provided the expertise and support they require (14). We know that an important part of creating an integrated care team is a defined identity. A socially constructed identity helps to mobilize and create shared ownership with diverse members which helps a team to run smoothly (12). Best and Williams (12) identified several things that enable collaborative identity: open-mindedness, communication and education, clear organization and structure of the new team, goal congruence, profession-specific mentoring and training, understanding the role of others, more diversity in the team.

A new clinical innovation, the Pathway for Eating Disorders and Autism developed from Clinical Experience (PEACE) team, decided to utilize brief huddles as an implementation technique. Research suggests that up to 37% of eating disorder patients have comorbid autistic traits (15). PEACE was formed as a direct result of the high level of comorbidity and the evident lack of response to traditional eating disorder treatment for this subgroup of patients (16, 17). It was apparent that this patient group needed a different treatment approach and with no current treatment guidelines available, the PEACE Pathway was innovated. PEACE is a care pathway with the aims of specifically supporting autistic patients with their eating disorder recovery, as well as their cares and clinicians (18). It is currently being piloted in South London and Maudsley NHS eating disorder services and PEACE resources and support materials are available freely online. For full details of PEACE implementation, see Tchanturia et al. (18) and for free materials visit our website: peacepathway.org.

Based on the apparent transferability of huddles [3, (4)], the aim of introducing huddles for this care pathway was: to improve team communication; to support efficient and effective, higher-level care; and to increase patient safety in a large specialist eating disorder (ED) treatment service. This patient groups' resistance to typical eating disorder treatment is a cause for safety concern, with the high mortality rates seen in eating disorder patients (19). Not all ED patients are autistic, so huddles aimed to be brief and weekly to fit with the needs of the Multidisciplinary team MDT they were supporting: evidence suggests tailoring huddles makes the benefits more pronounced (8). Whilst we can infer from our research that over the past 18 months that care has become more efficient and effective as the length of admission of autistic patients has decreased significantly (20), we need to evaluate the role of huddles in this context in order to see if they have a beneficial role in providing higher-level care.

Due to the complexity of EDs and their high medical risk, there is often involvement from different clinical disciplines such as nursing, psychology, dietetics and occupational therapy, making up the MDT. Improving communication and providing efficient and effective care was thought important as research and naturalistic observations provided evidence that autistic people fare far worse in standard eating disorder treatment than those who have low levels of autistic traits (16, 17). With approximately more than 80 members of the MDT, good communication and teamwork is a key element for implementing clinical innovation within a large service.

This study aimed to evaluate the benefits of the piloting use of MDT huddles in eating disorder treatment settings in providing a higher level of care for autistic patients with eating disorders.

## Methods

### Design and Period of Study

**Figure 1:**
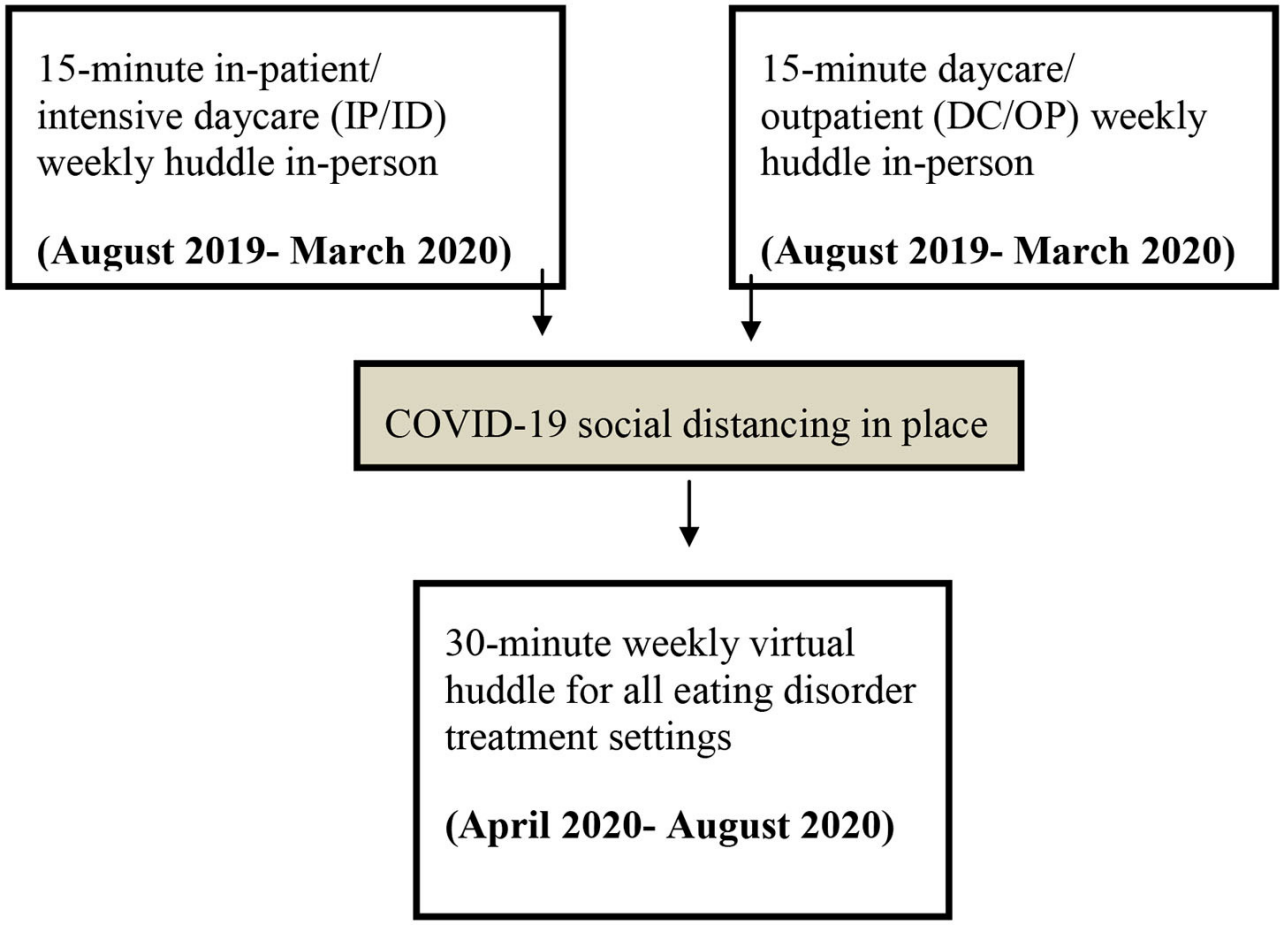
A flow diagram showing the huddle structure over time.

The implementation of the pathway took place over a national, specialist ED service, which was made up of two different sites: an inpatient/intensive daycare program (IP/ID) and a regular daycare/outpatient program (DC/OP). Due to the geographical distance between the two sites, initially in August 2019, it was decided that each site would have its own face-to-face huddle. Due to COVID-19 restrictions, in April 2020 these two huddles were combined to make on virtual 30-min huddle (see Figure 1 for a flow diagram showing huddle structure over time). Both settings were clinical services for adults (ages 18+).

The inpatient setting was made up of 18 beds, the intensive daycare program saw 10 patients at capacity, the regular daycare program saw 10 patients at capacity and the outpatient service saw ~500 patients per year at capacity. Of these eating disorder patients, research estimates that up to 37% are autistic or have high autistic features (15).

### Huddle Structure

Both face-to-face huddles lasted 15 min each taking place on different days to allow facilitators to attend both. The facilitators, in this case, were the pathway Principle Investigator (KT) and the Pathway Project Manager (KS). Following Plan, Do, Study, Act (PDSA) quality improvement cycles allowed frequent review of all aspects of implementation, including the huddles (21). The agenda of the huddle started unstructured, to see how the space was utilized and this was dependent on the feedback taken in the evaluation process. However, it always ended in a short evaluation. Toward the end of a year-long evaluation, the structure evolved into fixed pattern: general updates from the PEACE pathway implementation, specific feedback from clinicians on current PEACE patients, any other business and evaluation. Due to COVID-19, the huddles also had to be reviewed, with the last 4 months of huddles taking place virtually. This allowed flexibility in facilitator attendance and the two 15-min huddles, previously separate due to geographical location, merged into one 30-min huddle. With longer huddles, the time was often utilized at the start in the form of a short presentation on an adaptation or evaluation of the pathway. For example, if a new resource had been developed, a clinician would present it to the huddle, allowing clinicians to ask questions before it was rolled out and to open a discussion to get feedback for further development. Another presentation on the evaluation of these resources could then be presented after piloting it for a month.

### Participants

All members of the MDT were invited to join the weekly face-to-face huddles in their respected sites, and then after COVID-19 all to the same virtual huddle. Attendance was not mandatory, and attendees were welcome to come and go as their availability allowed, this became more relevant when the virtual huddles were extended to 30 min. Ethical approval for the study was obtained from South London and Maudsley NHS Foundation Trust (2019-004) as part of a service development project.

### Setting

The face-to-face huddles took place in the meeting room or the conference room at each respective site. These rooms both have seats available for up to 20 people and a dedicated huddle whiteboard for evaluation. After COVID-19, the huddles used a virtual conferencing program with a larger capacity.

### Data Collection and Analysis

The evaluation was collected in the form of a short 3-question survey weekly after each huddle. When huddles were face-to-face (pre-COVID-19), this was collected informally on a whiteboard where the three questions were written on the board and each attendee wrote their individual feedback underneath each other's comments after each face-to-face huddle. After the introduction of the virtual huddles, the same three-questions were sent out as a survey *via* email straight after the huddle had ended to each attendee for anonymous feedback.

The three evaluation questions were: 1. “How useful was the huddle?” (attendees were asked to mark /100 on a scale), 2. “What went well?,” and 3. “What could be improved?” Question 2 and 3 were open-ended questions. All attendees were encouraged to provide feedback at the end of each huddle.

Authors KS and KT read and reread qualitative data to ensure familiarity with the subject matter. Qualitative content analysis was then conducted on the data response to the two open-ended questions to identify themes (22). KS and KT independently reviewed each sample and proposed variables/themes for the analysis of each question, they then agreed on final variables/themes for each question response and coded each sample independently. The content of these codes in responses to each question was then calculated.

## Results

### Question 1: How Useful /100 Was the Huddle?

In total, 283 responses were collected with 88 from the IP/ID huddles, 65 from the DC/OP huddles and 103 from the virtual huddles (Table 1).

**Table 1:** A table showing the N, M, and Mode weekly responses in each huddle forum to “How useful /100 was the huddle.”

**Huddle**	**Number**	**Mean**	**Mode**
All	283	84	80
Inpatient/ Intensive Daycare	88	85	80
Daycare/Outpatient	65	81	80
Virtual	103	87	100

### Question 2: What Went Well in the Huddle?

**Figure 2:**
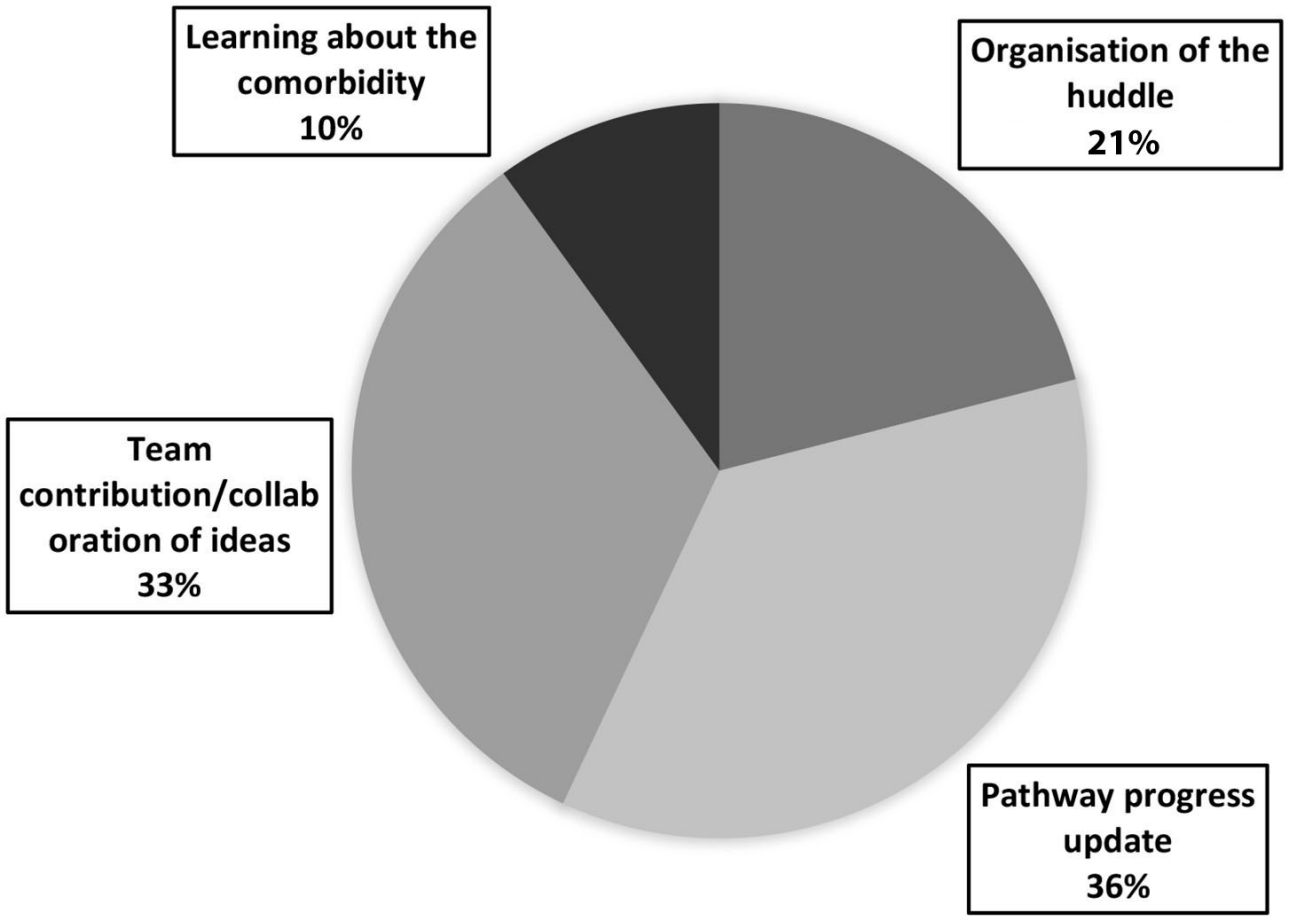
A pie chart representing the overall themes of responses to question 2 “what went well?”.

In total, 240 responses were collected to question 2: “What went well?” This was either collected on the whiteboard after a huddle or *via* the online survey collected after the huddle. Content analysis of the responses identified four distinct themes: organization, pathway progress updates, team contribution/collaboration, learning about the comorbidity (see Figure 2 for a pie chart demonstrating theme representation in responses to question 2; see Table 2 for a table demonstrating the frequency and percentage representation of each theme across the different huddles; Examples of each theme and subtheme can be found in Table 3).

**Table 2:** A table showing % themes identified from responses across the huddles to question 2 “what went well in the huddle.”

	**All responses to Q 2**	**Theme 1: pathway progress updates**	**Theme 2: team contribution/ collaboration of ideas**	**Theme 3: organization of huddle**	**Theme 4: learning about the comorbidity**
All	240 (100%)	87 (36%)	79 (33%)	51 (21%)	23 (10%)
Inpatient/ Intensive Daycare	78 (32.5%)	14 (18%)	31 (40%)	21 (27%)	12 (15%)
Outpatient/daycare	64 (26.67%)	25 (39%)	21 (33%)	16 (25%)	2 (3%)
Virtual	98 (40.83%)	48 (49%)	27 (28%)	14 (14%)	9 (9%)

**Table 3:** A table showing example quotes from each theme and subtheme identified from responses to question 2 “what went well in the huddle.”

**Theme**	**Subtheme**	**Clinician quotes**
Theme 1: pathway progress updates	Knowing about future events and dates Being informed about successes and challenges of the pathway Enjoyment of short presentations	“*Exciting to hear about future plans”* *“Keeping up to date with changes”* *“Hearing about the food provision problem”* *“Great to be updated on positive news”* *“A very interesting, useful presentation”* *“Very informative presentation on sensory processing. good links to clinical practice”*
Theme 2: team contribution/collaboration of ideas	Generating new ideas together General discussions around adaptations and implementations Team-work ethos	“*Joint thinking”* *“Lots of useful ideas generated”* *“discussing potential tools to support ASD patients”* *“website discussion and brainstorming generated good ideas”* *“Involvement from different services, and everyone's willingness to listen”* *“very inclusive team with members from all related disciplines”*
Theme 3: organization of the huddle	Attendance Structure/Facilitation/Timing and Efficiency Enjoyment of virtual structure	“*Lots of people joined”* *“MDT presence”* *“Smooth and to the point”* *“Well run and structured”* *“Good organization, competent, enthusiastic”* *“Creativity around remote working”* *“Keeping PEACE live throughout the recent upheavals”*
Theme 4: learning about the comorbidity	Learning about adaptations Learning about the needs of the stakeholders Learning about relevant research application	“*It is helpful to hear about the different techniques to use”* *“information giving on opportunities for patient and carers”* *“Understanding more about our clients”* *“very informative- good to know about patient presentations”* *“very informative presentation on sensory processing. good links to clinical practice”* *“Sharing info from conference”*

Theme 1: Pathway progress updates. Feedback regarding appreciation for being kept up to date made up 36% or the responses to question two, the largest theme. Subthemes identified included: knowing about future events and dates, being informed about successes, and challenges the pathway is currently facing (including funding, catering, conferences, informing the team of new resources and PEACE dissemination). Additionally, once the huddles became virtual, a subtheme of updating on progress was identified through expressions of appreciation for the short presentations on the pathway. Theme 1 made up 18% (*N* = 14) of IP/ID responses, 39% (*N* = 25) of DC/OP responses, and 49% (*N* = 48) of virtual responses.

Theme 2: Team contributions/collaboration was expressed by 33% (N=) of responses to question 2. Several subthemes included: generating new ideas together, general discussions about adaptation and implementations, team-work ethos. Another subtheme identified which was only present in the daycare/outpatient and virtual huddle feedback was the discussion of specific cases. Theme 3 made up 40% (*N* = 31) of IP/ID responses, 33% (*N* = 21) of DC/OP responses, and 28% (*N* = 27) of virtual responses.

Theme 3: Organization of the huddle. Positive feedback for how the huddles were organized made up 21% (*N* = 51) of the “what went well” responses. Subthemes included: attendance, structure, facilitation, timing and efficiency. An additional subtheme was identified from the virtual huddle feedback: enjoyment of virtual structure. This theme made up 27% (*N* = 21) of IP/ID responses, 25% (*N* = 16) of DC/OP responses, and 14% (*N* = 14) of virtual responses.

Theme 4: Learning/educative. The final theme identified was the smallest theme and made up 10% of response data to question 2. Subthemes identified here included learning about adaptations, learning about the needs of the PEACE stakeholders and learning about relevant research application. Theme 4 made up 15% (*N* = 12) of IP/ID responses, 3% (*N* = 2) of DC/OP responses, and 9% (*N* = 9) of virtual responses.

### Question 3: What Could Be Improved?

**Figure 3:**
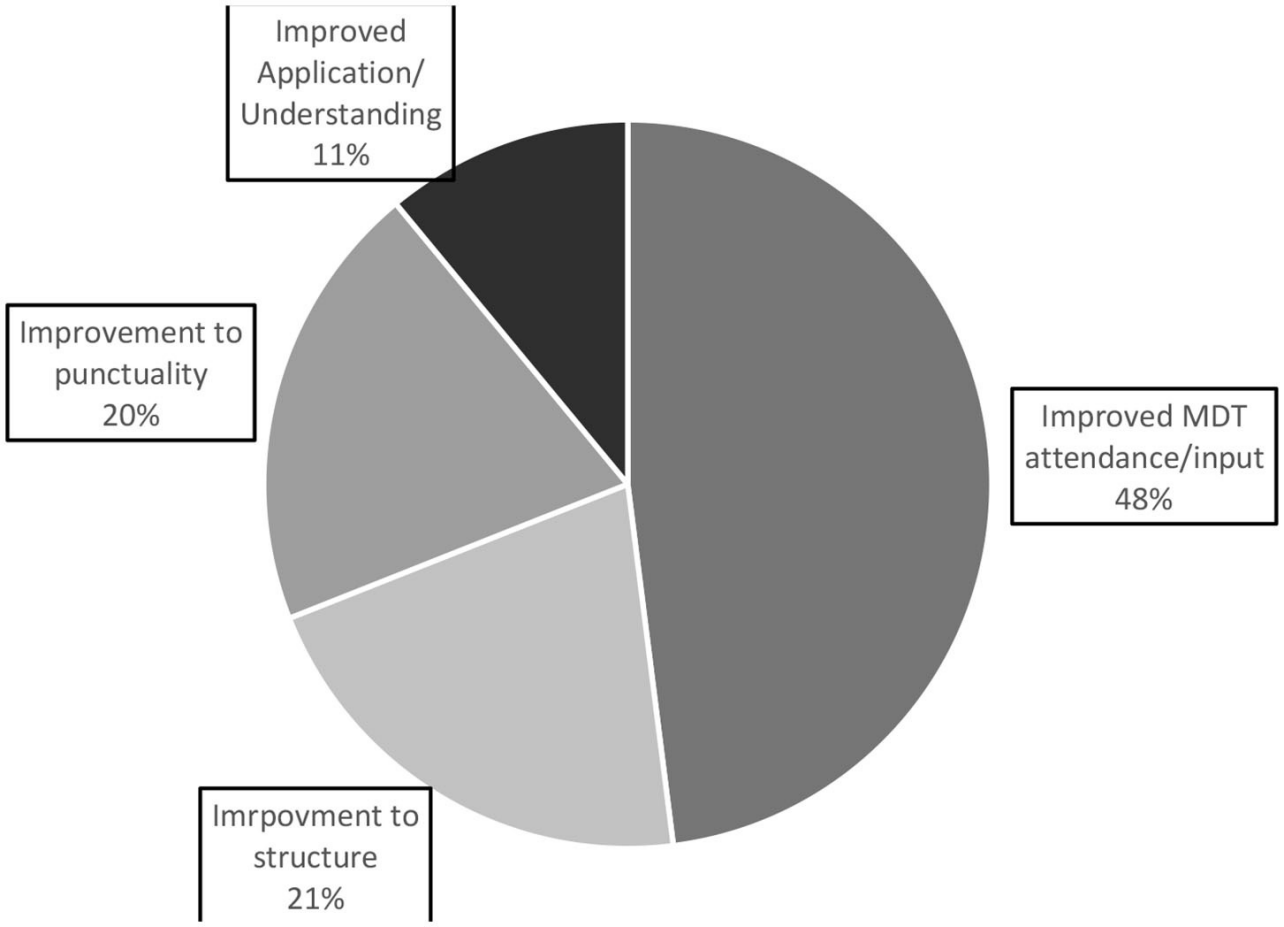
A pie chart representing the overall themes of responses to question 3 “what could be improved in the huddle?”.

In total, 117 responses were collected from question three “What could be improved?” After cleaning the data set of responses that were irrelevant to our research question (i.e., personal circumstance, writing “N/A” or regarding technical issues), 61 responses were coded using a content analysis approach (22); (see Figure 3 for a pie chart demonstrating total theme representation in responses to question 3; see Table 4 for a table demonstrating the frequency and percentage representation of each theme across the different huddles; examples of each theme and subtheme can be found in Table 5).

**Table 4:** A table showing % themes identified from responses across the huddles to question 3 “what could be improved in the huddle.”

	**All responses to Q 3**	**Theme 1: improved MDT attendance/ input**	**Theme 2: improvements to structure**	**Theme 3: punctuality**	**Theme 4: improved application and understanding**
All	61	29 (48%)	13 (21%)	12 (20%)	7 (11%)
Inpatient	33	22 (66%)	2 (6%)	2 (6%)	7 (21%)
Outpatient/daycare	9	1 (3%)	2 (22%)	6 (67%)	0 (0%)
Virtual	19	6 (21%)	9 (47%)	4 (21%)	0 (0%)

**Table 5:** A table showing example quotes from each theme and subtheme identified from responses to question 3 “what could be improved about the huddle?”

**Theme**	**Subtheme**	**Clinician quotes**
Theme 1: improved MDT attendance/input	Attendance Input	“*attendance I wish more of the MDT could hear it!!!!”* *“Even more staff attendance”* *“More input from attendees”* *“some people did not speak—please join in!”*
Theme 2: team improvements to structure	Pre-agenda Focus Action points	“*Email agenda prior to snapshot”* *“Some conversations that are not truly relevant for the larger group”* *“was not really sure what we suggestions we came out of the huddle with”*
Theme 3: punctuality		“*Timing”* *“Finishing on time”*
Theme 4: improved application and understanding	Learning Improving Application	“*learning about what else can be done for our patients ”* *“training for newbies!”* *“Kitchen noise and foot traffic during mealtimes”* *“Chance to apply this in an admission”*

Theme 1: Improved MDT attendance/input. By far the largest theme of the responses was about improving MDT attendance/input. The majority of these responses were in terms of increasing attendance, where a few were on increased involvement from those attending. This theme made up 66% (*N* = 22) of IP/ID responses, 3% (*N* = 1) of DC/OP responses, and 21% (*N* = 6) of virtual responses.

Theme 2: Improvements to structure. This theme made up 21% of overall responses for “what could be improved.” Reoccurring suggestions included providing more information before such as a pre-agenda, keeping focus in the meeting and creating action points. This theme made up 6% (*N* = 2) of IP/ID responses, 22% (*N* = 2) of DC/OP responses, and 47% (*N* = 9) of virtual responses.

Theme 3: Punctuality. Making up 20% of response data, punctuality was indicated as something that could be improved in the huddles with staff wanting to finish the meetings on time. Theme 3 made up 6% (*N* = 2) of IP/ID responses, 67% (*N* = 6) of DC/OP responses, and 21% (*N* = 4) of virtual responses.

Theme 4: Improving application and Understanding of the pathway. This final theme made up 11% of overall feedback for question 3 “What could be improved?” Sub-themes for this were more learning and improving application. This theme made up 21% (*N* = 7) of IP/ID responses, 0% (*N* = 0) of DC/OP responses, and 0% (*N* = 0) of virtual responses.

## Discussion

This paper looked at how useful weekly huddles are as an implementation technique in implementing an innovative pathway in a multi-disciplinary mental health service. A total of 283 responses evaluating huddles were collected over a 12-months period, and huddles were assessed as useful on average 84/100. Looking at what went well in the huddles, feedback suggested several benefits; keeping the teams updated on the pathway's progress, collaborations and MDT discussions, and education. Feedback suggests huddles were well-organized and well structured. In terms of what could be improved with the weekly huddles, themes identified in the feedback included the need for improved MDT attendance/input, Improvements to structure, improvements in punctuality and improved fostering understanding and application.

### Question One: How Useful Was the Huddle?

Overall, all three huddle “types” were well-received, but It is interesting to consider why Virtual huddles, which were twice a long and combined IP/ID and DC/OP staff, were rated higher in usefulness than in-person huddles. We can understand from the literature, that the theoretical underpinnings of huddles may have better been supported in this virtual format.

The virtual huddles were reintroduced in a time of uncertainty (COVID-19 lockdown) and responses showed that people appreciated some form on consistency and opportunity to meet with their colleagues: “Great to catch up with everyone,” “keeping in touch with PEACE,” “good to check in on everyone now we are remote working,” and “Keeping PEACE alive through recent upheavals.” This suggests that some form of shared professional identity may have been built, resulting in a sense of belonging and group ownership, as is common with the use of huddles (12). As a result of COVID-19, this professional identity would have been disrupted with many staff working from home, virtual huddles may have given an opportunity to reinstate that identity, leading to a higher level of usefulness. Furthermore, this shared, professional identity could have been strengthened with the introduction of short presentations, which would have increased communication and education (12). Virtual huddles also bridged the gap between the two huddles: “I'm really impressed by how well the PEACE huddles have transitioned to virtual meetings- I think if anything they're better as we get more attendance/ it's nice having combined perspectives from the out/day/inpatient teams.” “Hearing what happened in other parts of the service,” “joint meeting across the service is great,” “The attendance is still really good, better than when we did in-person huddles?” and “I always enjoy the updates from different staff and services,” further emphasizing the shared identity and the role regular huddles serve in nurturing that. From the observation of the principal investigator (corresponding author), the huddles created a space for shared knowledge, developing culture and confidence to implement the PEACE pathway.

### Question Two: What Went Well in the Huddle?

We examined the themes identified to understand how huddles were received as an implementation technique. We wanted to know if the themes supported the theoretical underpinnings of huddles for this innovative clinical pathway and if huddles are transferable as implementation techniques.

Our four themes identified were: pathway progress updates, team contribution/collaboration of ideas, organization, and learning about the comorbidity. Theoretical underpinnings of huddles have been identified as promoting benefits to attendees such as teamwork, communication, education and training, and shared professional identity (12).

Theme one “pathway progress updates” made up 36% of total responses. From the literature, we can infer that our first theme relates to the shared professional identity around the new pathway, and this identity was created and dynamically constructed through participation in huddles (12). Attendees felt invested in the pathway, meaning that updates specifically around the pathway progress were seen as highly important. Furthermore, this theme featured most strongly in virtual huddles (49%) which supports this interpretation. Professional identity is likely to diminish and become more uncertain through remote working during the COVID-19 pandemic and because we joined the two huddles together, prompting shifts in identity constructions. Also looking at the theoretical underpinnings of a huddle, we can see this theme of updates incorporates communication. Enhanced communication is a desired benefit of huddles leading to team cohesion as failure to communicate has often been identified as the reason for medical errors (23). The theme confirms the theoretical underpinnings of huddles and demonstrates the potential transferable nature of huddles as a valuable technique or strategy in implementing and integrating an innovative pathway.

The second theme, “team contribution/collaboration of ideas” made up 33% of total responses. Again, looking at the theoretical underpinnings of huddles, we can see alignment in the importance of the role of teamwork, communication, and shared professional identity. These themes made up the largest part of IP/ID huddles responses (40%), higher than in the other two huddles (DC/OP- 33%, virtual- 28%), probably because the IP/ID programmes require an MDT given the higher medical risk to patients. The DC/OP services run more independently with some patients only seeing one member of staff in OP, meaning that team cohesion from an MDT may not have existed before joining the huddles. A separate sub-theme of the value of specific case study discussions was identified and was not present in the other groups. This suggests that the DC/OP huddle was utilized more as group supervision for individual cases where attendees had an opportunity to share ideas and develop consistency in treatment implementation. Huddles, therefore, created teamwork which creates cohesion. We know that without teamwork and cohesion bad things happen to patients.

Data from the IP/ID produced subthemes of generating ideas together, general discussions about adaptation and implementations and team-work ethos. The IP/ID programmes already have allocated spaces to discuss shared cases collaboratively and therefore clearly utilized huddles differently. This demonstrates the transferable nature of huddle as an implementation technique, and how it adapts depending on the needs of the group. We know that structuring the huddle to suit the needs of the attendees brings pronounced benefits (8). In this case, the group and feedback determined the structure. We need to mindful that the group's determination of how a huddle is utilized may clash when combining groups. However, with the weighting of this theme focused on teamwork ethos, it would seem the benefits of combining the two groups outweighed the balancing of differing needs.

Theme three “organization of the huddle” made up 21% overall of feedback on what went well in the huddle. This was made up of several subthemes: attendance, structure, facilitation, timing and efficiency. An additional subtheme from the virtual huddle feedback was enjoyment of virtual structure. With attendance being the largest sub-theme of “organization of the huddle” we can see again how valuable that shared identity (12) is to the huddle attendees. This themes also demonstrates the value of the structuring the huddle to suit the needs of the attendees for pronounced benefits (8). We can also see with the introduction of the subtheme “enjoyment of the virtual structure” that attendees valued combining the huddles to emphasize the teamwork and shred professional identity as well as the role of education as a theoretical underpinning for huddles (12) with the introduction of brief, educative presentations.

Theme four of question 1: “what went well” was “learning/educative” and made up 10% of responses. Subthemes identified included learning about adaptations, learning about the needs of the PEACE stakeholders and learning about relevant research application. This was identified most in IP/DC (15%, then virtual (9%) and then 3% for DC/OP. When looking at the underpinning themes of huddles again, education and communication are both highlighted, which suggests that the huddles are a valuable implementation technique due to their transferable nature.

### Question Three: What Could Be Improved?

For question 3: “what could be improved,” four themes again were identified. These were: improved MDT attendance/input, Improvements to huddle structure, punctuality and application & understanding. This was useful in seeing how elements of huddles were perhaps not transferable or highlighting aspects to attend to when using huddles as implementation techniques.

Theme 1 was “improved MDT attendance/input.” This was by far the majority of IP/ID responses, making up 66% what could be improved. This reflects the importance of MDT treatment cohesion, how the inpatient model relies heavily on the MDT and how clinical innovations can be hard to implement if the attendance/input is not representative of all disciplines. It suggests that the huddle could be more useful if there was greater representation from diverse disciplines: “Attendance- I wish more of the MDT could hear it!,” “More disciplines,” and “Other disciplines to attend.” This is contrasted to only 3% of DC/OP identifying this because as already discussed, multidisciplinary cohesion is not a norm. The virtual huddle responses predictably represented both with 22% of responses suggesting there was a lack of MDT representation. Again, this theme could be interpreted as supporting how the team value the shared identity and teamwork, and how they want more attendees and disciplines to join (12).

Theme 2 was “Improvements to structure,” making up 21% of total responses. This was considerably higher for the virtual huddles (47 vs. 6% and 22%) which reflects the novel structure of the virtual huddles and the need to refine the structure as these incorporated two different groups. The refinements happened over time using the PDSA format.

This theme also covered content, including suggestions on how to improve huddles with “Email agenda prior” being the most popular. This was implemented during the virtual huddles. Other responses included the need to keep a focus in the huddles: “Some conversations that are not truly relevant for the larger group,” as well as creating action points: “Was not really sure what suggestions we came out of the huddle with.” This feedback makes it clear that in implementing this type of huddle, a pre-agenda (and sticking to it) is useful for keeping focused. Creating action points after each huddle is needed, perhaps documenting these in the minutes. However, some of this data might arise from the necessary combining of two huddles, as there may be information shared which is only relevant to one treatment program, and therefore not “for the larger group.” Further structure refinements could improve this.

Theme 3 for question 3 was “Punctuality,” and it made up 20% of responses. This was particularly noted in DC/OP huddles where 67% of responses to question 3 were in regards to running to time. This could be due to the fact that these huddles would often be used to discuss individual cases more, perhaps leading to a looser agenda and consequently running overtime.

Theme 4 for question 3 on how the huddles could improve was “improved application and understanding,” representing 11% of responses. Interestingly, this theme was only found in IP/ID responses (21%). This could be due to research suggesting that patients with the comorbidity have more severe clinical presentations and longer inpatient admissions (16, 17), perhaps meaning the IP/ID clinicians encountered this comorbidity more and that application and understanding was more important. The fact that this was not highlighted in the virtual huddles feedback could mean that the introduction of presentations satisfied this need.

### Future Research and Limitations

Future research could examine the option of implementation of a virtual huddle or face-to-face, determining which format is best received in different clinical settings. Examining the application of huddles to other clinical implementation pathways would be useful. Another direction could be to evaluate care effectiveness and efficiency as a result of regular huddles.

One limitation of this study is that in the face-to-face huddles, feedback was not completely anonymous. Although the facilitators did not monitor the identity of anyone writing feedback on the whiteboard, other team members would have seen what was being written. This could have impacted what attendees wrote down due to social perception and wanting to be in the in-group. Furthermore, with the scale on question one, attendees would often cluster their score around the first score noted down. Although this was a pilot study and to ensure that the maximum amount of feedback was received meant making the evaluation as time-efficient and straightforward as possible. Future research may want to make this face-to-face data anonymous. A further limitation of this study is its implementation to a single service, giving a limited scope for the data as well as a potential bias. However, as previously mentioned this was a pilot study, which allows for other groups to adopt practice and further evaluate effectiveness.

## Conclusions

Overall, the data suggest that weekly huddles for quality improvement implementation were well-received and useful, both face-to-face and virtually. The weekly evaluation suggested that huddles are a useful implementation tool in creating a shared identity, teamwork culture and space for education in innovation implementation. This highlights the value of huddles and demonstrates their transferability (12). The longer, virtual huddles were potential better received due to it reintroducing a sense of structure, shared identity and learning during the COVID-19 pandemic. Furthermore, they bridged the gap between different treatment teams and allowed more detailed updates. Pathway progress updates were well-received in all the huddles, with team contributions and attendance being most valuable in inpatient/intensive daycare huddles. Attendees appreciated when the huddles were well-structured and lead by an agenda, as well as keeping to the allotted time. Pre-agendas, agendas and brief presentations were looked on favorably and helped to keep the huddle focused. The data suggested that the huddle may have different benefits for each treatment team, with outpatient clinicians, often working individually, enjoying using the huddle time as group supervision and the inpatient/intensive daycare team looking for more opportunity for understanding and application. Huddles in the context of novel clinical pathway developments are valuable in creating a shared identity, culture, and educative space.

## Data Availability Statement

The raw data supporting the conclusions of this article will be made available by the authors, without undue reservation.

## Ethics Statement

The studies involving human participants were reviewed and approved by Ethics 2019-004 from South London and Maudsley NHS Foundation Trust governance committee. Written informed consent for participation was not required for this study in accordance with the national legislation and the institutional requirements.

## Author Contributions

KS and KT collected, analyzed the data, and wrote the paper. KT is the principal investigator of the PEACE pathway project, developed the study protocol, and supervised team. KS is a project manager collecting the data and managing day to day activities in the project. All authors contributed to the article and approved the submitted version.

## Conflict of Interest

The authors declare that the research was conducted in the absence of any commercial or financial relationships that could be construed as a potential conflict of interest.
